# Hypoxia-induced lncRNA PDIA3P1 promotes mesenchymal transition via sponging of miR-124-3p in glioma

**DOI:** 10.1038/s41419-020-2345-z

**Published:** 2020-03-03

**Authors:** Shaobo Wang, Yanhua Qi, Xiao Gao, Wei Qiu, Qinglin Liu, Xiaofan Guo, Mingyu Qian, Zihang Chen, Zongpu Zhang, Huizhi Wang, Jianye Xu, Hao Xue, Xing Guo, Ping Zhang, Rongrong Zhao, Gang Li

**Affiliations:** 1 Department of Neurosurgery, Qilu Hospital of Shandong University, 107 Wenhua Xi Road, 250012 Jinan, Shandong Province China; 2 Institute of Brain and Brain-Inspired Science, Shandong University, 107 Wenhua Xi Road, 250012 Jinan, Shandong Province China; 3 Shandong Key Laboratory of Brain Function Remodeling, 107 Wenhua Xi Road, 250012 Jinan, Shandong Province China; 4 Department of Interventional Neuroradiology, Beijing Neurosurgical Institute and Beijing Tiantan Hospital of Capital Medical University, Beijing, China

**Keywords:** Cancer genomics, CNS cancer

## Abstract

Hypoxia is a critical factor in the malignant progression of glioma, especially for the highly-invasive mesenchymal (MES) subtype. But the detailed mechanisms in hypoxia-induced glioma MES transition remain elusive. Pseudogenes, once considered to be non-functional relics of evolution, are emerging as a critical factor in human tumorigenesis and progression. Here, we investigated the clinical significance, biological function, and mechanisms of protein disulfide isomerase family A member 3 pseudogene 1 (PDIA3P1) in hypoxia-induced glioma MES transition. In this study, we found that PDIA3P1 expression was closely related to tumor degree, transcriptome subtype, and prognosis in glioma patients. Enrichment analysis found that high PDIA3P1 expression was associated with epithelial-mesenchymal transition, extracellular matrix (ECM) disassembly, and angiogenesis. In vitro study revealed that overexpression of PDIA3P1 enhanced the migration and invasion capacity of glioma cells, while knockdown of PDIA3P1 induced the opposite effect. Further studies revealed that PDIA3P1 functions as a ceRNA, sponging miR-124-3p to modulate RELA expression and activate the downstream NF-κB pathway, thus promoting the MES transition of glioma cells. In addition, Hypoxia Inducible Factor 1 was confirmed to directly bind to the PDIA3P1 promotor region and activate its transcription. In conclusion, PDIA3P1 is a crucial link between hypoxia and glioma MES transition through the PDIA3P1-miR-124-3p-RELA axis, which may serve as a prognostic indicator and potential therapeutic target for glioma treatment.

## Introduction

Human brain gliomas represent 75% of malignant primary brain tumors in adults^1^. Although treatments including maximal safe resection, radiotherapy, and adjuvant temozolomide have evidently improved the outcomes of glioma patients, they still lag behind those of other tumors^2,3^. Due to the emerging identification of biomarkers which affect the biological features of tumors, glioma genotype can be more informative than histological phenotype in providing a more accurate diagnosis, reliable prognosis, and treatment strategies in early phase trials^4–6^.

Long non-coding RNAs (lncRNAs) are transcripts without protein-coding function, which account for 98% of the human transcriptome^7^. To date, numerous studies have demonstrated that lncRNAs work as novel regulators of transcriptional and epigenetic networks^8–11^. Moreover, many lncRNAs were found to be aberrantly expressed in tumors and to play critical roles in tumor progression^12,13^. Among them, pseudogenes are defined as genomic loci that resemble to their coding homologs and lack of translation into functional proteins^14^. However, increasing evidence has revealed the multifunctional character of pseudogenes in contribution to tumor progression^15–17^.

Hypoxia is a hallmark of gliomas^18^. Lack of oxygen alters the expression properties of tumor cells, which activate downstream genes to facilitate tumor growth, angiogenesis, and metastasis^19^. Strong evidence has shown that hypoxia is closely correlated with the renewal capacity maintenance of glioma stem cells (GSCs) and the mesenchymal (MES) subtype in transcriptome sequencing^20,21^. In addition, our previous studies demonstrated that hypoxia is a critical factor in tumor growth, metastasis, and immune microenvironment modification^22,23^. In this study, we analyzed the microarray data of U87MG glioblastoma cells cultured under normoxic (21% oxygen) or hypoxic (1% oxygen) conditions from the Gene Expression Omnibus (GEO), and identified protein disulfide isomerase family A member 3 pseudogene 1 (PDIA3P1), a 2099-bp segment mapping to chromosome 1q21.1, which has been reported highly expressed in HCC and OSCC, upregulated under hypoxic conditions^24,25^. However, the regulation of gene expression and functional mechanisms of PDIA3P1 require further investigation.

Further analysis using The Cancer Genome Atlas (TCGA) and the Chinese Glioma Genome Atlas (CGGA) database showed that high PDIA3P1 expression represents a more malignant tumor type and results in poorer outcomes in glioma patients. PDIA3P1 overexpression or knockdown changed the migration and invasion capacity of glioma cells. This occurred by sponging of miR-124-3p to modulate the expression of RELA, and activating the downstream NF-κB pathway to promote MES transition in glioma cells. Further studies revealed that the HIF1 heterodimer directly bound to the hypoxia response element (HRE) of the PDIA3P1 promoter to facilitate its expression under hypoxic conditions. Collectively, this study demonstrated that PDIA3P1 is a crucial link between hypoxia and glioma MES transition, thus it may serve as a potential candidate for predicting prognosis and as a target for therapy in glioma patients.

## Materials and methods

### Microarray dataset

The public U87MG glioblastoma cell line microarray gene profiling dataset GSE45301 was downloaded from the Gene Expression Omnibus (GEO) using the Illumina Human HT-12 V3.0 platform. RNA sequencing data of cancer tissue samples were obtained from TCGA (https://cancergenome.nih.gov) and CGGA (http://www.cgga.org.cn/). The cut-off value between high and low PDIA3P1 expression was set as the expression level of a median sample. Clinical attributes were download from the TCGA dataset.

### Enrichment analysis

Biological process (BP) of Gene Ontology (GO), Kyoto Encyclopedia of Genes and Genomes (KEGG), and correlation analysis of PDIA3P1 were performed on gene expression profiles available in the TCGA dataset. Differentially expressed genes (DEGs) were obtained using the DESeq2 package of R, and significantly variated genes were defined as |log2FC|≥ 2, *p* < 0.05. DAVID web tool (http://david.abcc.ncifcrf.gov/home.jsp) was used to enrich and analyze the DEGs. The association between PDIA3P1 expression and hallmark gene sets from the Molecular Signatures Database (MSigDB) were analyzed using gene set enrichment analysis (GSEA) software (http://software.broadinstitute.org/).

### Cell culture

Human glioma cell lines U251, U87MG, and A172 were purchased from the Chinese Academy of Sciences Cell Bank. Cells were maintained as previously described^26^. For cellular hypoxia induction, glioma cells were cultured in CO2 incubator (CB60, Binder) under 1% O_2_, 5% CO_2_, 37 °C for 72 h. Short tandem repeat profiling was used to authenticate all cell lines. The cells were confirmed as mycoplasma negative before use.

### RNA extraction and real-time quantitative PCR (RT-qPCR)

TRIzol (Invitrogen, Carlsbad, CA, USA) was used to extract total cell RNA in accordance with the manufacturer’s protocol. Reverse transcription and RT-qPCR was performed as previously described^22^. The primers used are shown in Supplementary Table 1. The relative expression levels were evaluated using the ΔΔCt method.

### Lentivirus, siRNA, microRNA, plasmid construction, and cell transfection

Human full length PDIA3P1 plasmids was selected and inserted into the pLVX-IRES-Puro vector for stable overexpression. The si-PDIA3P1, si-RELA, miR-124-3p mimics, miR-124-3p inhibitors, and negative controls were purchased from Genepharma (Shanghai, China). All sequences are listed in Supplementary Table 2. Plasmids were purchased from Bioscience (Jinan, China), and the human PDIA3P1 promoter sequence was obtained from the UCSC Genome Browser (http://genome.ucsc.edu/). For transfection, cells were seeded in 6-well plates overnight and transfected using Lipofectamine 3000 (Invitrogen, CA, USA) according to the manufacturer’s instructions. Cells were harvested for RT-qPCR and western-blot analysis 48 h after transfection.

### Dual-luciferase reporter assays

The dual luciferase reporter plasmids (pGL3-PDIA3P1 WT/MUT and pGL3-RELA WT/MUT), and PDIA3P1 promoter luciferase reporter plasmids were designed and synthesized by Bio-Asia (Jinan, China). HEK-293T cells were seeded in 96-well plates overnight (2 × 10^4^/well). For 3’UTR tests, the dual luciferase reporter plasmids (0.1 μg/well) were co-transfected with miR-Nc or miR-124-3p mimics (20 nM × 0.5 μl/well). For promotor testing, PDIA3P1 promotor luciferase reporter plasmids (0.1 μg/well) and pRL-TK plasmid (5 ng/well) were co-transfected with pENTER (0.1 μg/well) or the HIF1A overexpressing plasmid (0.1 μg/well) using Lipofectamine 3000 (Invitrogen, Carlsbad, CA, USA). PRL-TK was used as the internal control. Cells were subjected to luciferase activity analysis using a Dual-Luciferase Reporter Assay System (Promega, USA) following the manufacturer’s instructions.

### Biotin-labeled RNA pulldown assay

Biotinylated miR-124-3p and control miRNA (GenePharma, Shanghai, China) were transfected into U87MG cells and U251 cells. Cell lysates were collected 48 h after transfection, then incubated with M-280 streptavidin magnetic beads (Invitrogen) and 10 μL of yeast tRNA on a rotator at 4 °C overnight. The bound RNAs were purified by adding 750 μL of TRIzol (Invitrogen, Carlsbad, CA, USA) per sample and 250 μL of water to the input and the pull-down beads for further RT-qPCR analysis.

### Cell migration and invasion assays

Transwell assay were performed as previously described^10^. 3D tumor spheroid invasion assay was performed using the 96 well 3D spheroid BME cell invasion assay kit (3500-096-K, Trevigen, USA) according to manufacturer’s protocol. In brief, 2 × 10^5^ glioma cells were cultured in spheroid formation ECM for 72 h to generate tumor spheroids. Then, invasion matrix was added to the tumor spheroids. Tumor spheroids were photographed at 0-h, 24-h, 48-h, and 72-h under a Leica microscopy.

### Fluorescence in situ hybridization (FISH)

U87MG, A172, and U251 cells were fixed in 4% paraformaldehyde for 15 min, washed with PBS, treated with pepsin (1% in 10 mM HCl), and then dehydrated with 70%, 85%, and 100% ethanol. For denaturation, cells were air-dried and incubated with a 20 nM FISH probe in hybridization buffer at 73 °C for 5 min in a water bath. The hybridization was performed at 37 °C for 12 h. Finally, slides were washed, dehydrated, and stained with DAPI for detection. The RNA FISH probe was designed and synthesized by Genepharma (Shanghai, China), sequences are listed in Supplementary Table 3.

### Western blotting

The western blotting assay was performed as previously described^22^. The following primary antibodies were used: β-actin (Proteintech, 60008-1-Ig), Vimentin (Cell Signaling Technology, 5741), N-cadherin (Cell Signaling Technology, 13116), β-Catenin (Cell Signaling Technology, 8480), CD44 (Proteintech, 15675-1-AP), MMP14 (abcam, ab51047), Phosphorylated NF-κB p65 (S536) (Cell Signaling Technology, 3033), NF-κB p65 (Cell Signaling Technology, 8242), and HIF-1α (Cell Signaling Technology, 36169).

### Immunohistochemistry (IHC)

IHC assay was performed as previously described^27^. Following primary antibodies were used: anti-CD44 (Proteintech, 15675-1-AP), anti-Vimentin (Cell Signaling Technology, 5741), anti-NF-κB p65 (Cell Signaling Technology, 8242), and anti-Phosphorylated NF-κB p65 (S536) (abcam, ab86299).

### Chromatin immunoprecipitation (ChIP) assay

The binding sites of HIF-1α and HIF-1β heterodimer (HIF1A::ARNT) on the promotor region of PDIA3P1 were predicted using JASPAR (http://jaspar.genereg.net/). ChIP assay were performed using the Magna ChIP™ A/G Chromatin Immunoprecipitation Kit (17-10086, Millipore, USA) as previously described^10^. The following antibodies were used: anti-HIF-1α (Cell Signaling Technology, 36169) or anti-HIF-1β (Cell Signaling Technology, 5537). Co-precipitated DNA was quantified using PCR and RT-qPCR. The PDIA3P1 promoter primers used are shown in Supplementary Table 1.

### Animal studies

Luciferase labeled and stably transfected U87MG cells overexpressing PDIA3P1 or vector were injected into the brains of randomly grouped 4-week BALB/c nude mice (5 × 10^5^/mouse) to build the orthotopic xenograft model. Bioluminescence imaging was used to image the mouse brains every 5 days after glioma cell implantation. Next, we randomly chose 5 mice in each group and euthanized them on the same day (10 day). The brains were fixed with paraformaldehyde for further study. The remaining mice (5/group) were kept until death for survival analysis. All procedures that involved mice were approved by and under the requirements of the Animal Care and Use Committee of the Qilu Hospital of Shandong University.

### Statistical analysis

The cut-off value between high and low PDIA3P1 expression was set as the expression level of a median sample. Survival analysis was performed using the Kaplan–Meier method and comparisons were done using the log-rank test. Two-tailed *χ*^2^ test was used to estimate the association between PDIA3P1 expression and clinicopathological characteristics. Pearson correlation was used to evaluate the linear relationship between the expression of different genes. The one-way ANOVA test or Student’s *t* test were used for all other data comparisons using GraphPad Prism 7. All data are presented as the mean ± standard error (S.E.) and *P*-values < 0.05 were considered statistically significant.

## Results

### LncRNA PDIA3P1 was upregulated in hypoxia-treated glioma cells and correlated with poor prognosis

**Fig. 1:**
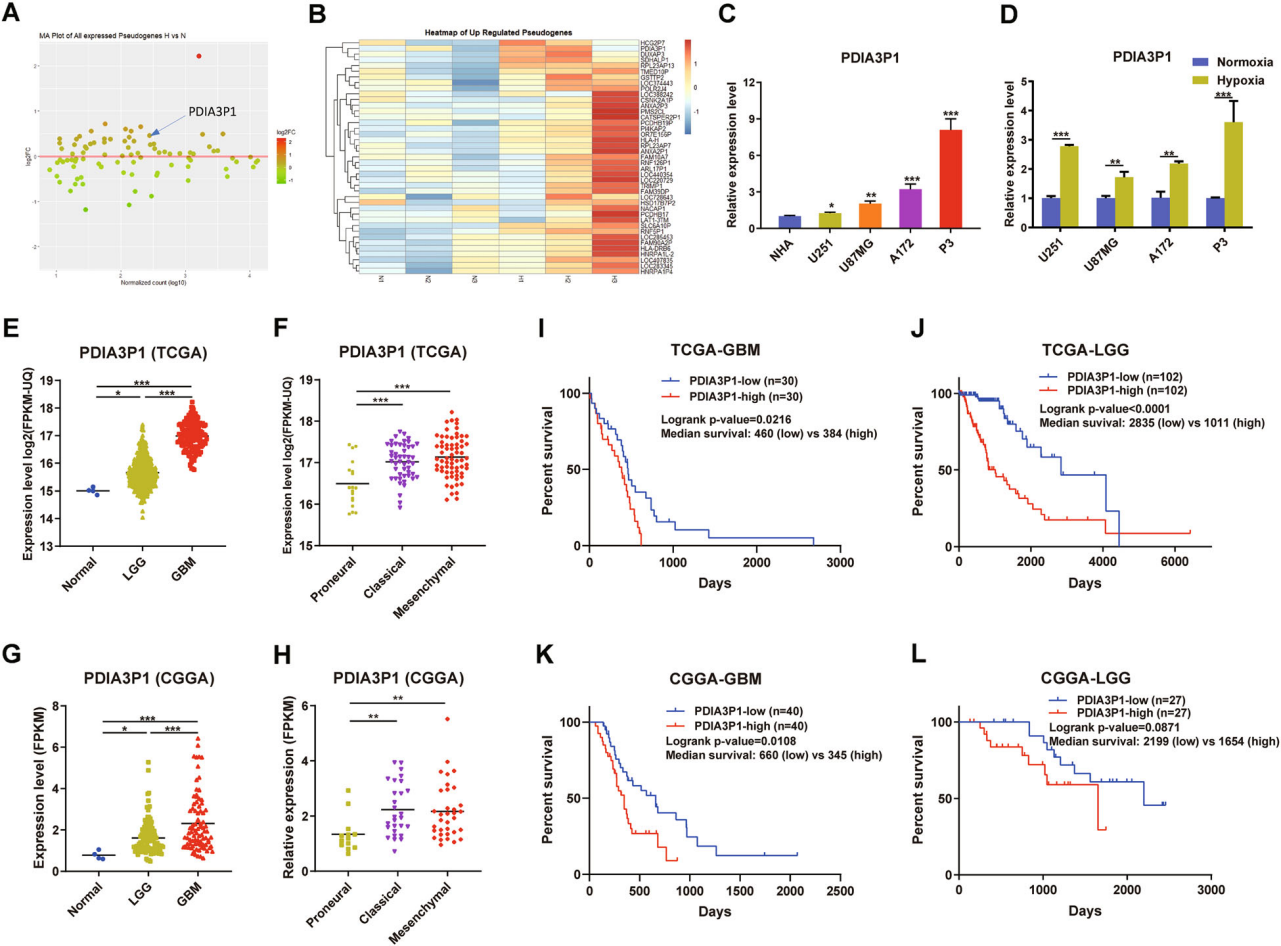
The pseudogene PDIA3P1 was upregulated in hypoxia-treated glioma cells and correlated with poor outcomes in glioma patients. **a** MA plot showing the alteration of pseudogenes in hypoxia-treated U87MG cells compared to their normoxia-treated counterparts. **b** Heatmap demonstrating the upregulated pseudogenes in hypoxia-treated U87MG cells. **c** Real-time quantitative PCR (RT-qPCR) evaluating the relative expression of PDIA3P1 in normal human astrocytes (NHA), glioma cell lines (U251, U87MG, and A172), and primary GBM cells (P3). **d** RT-qPCR testing the relative expression of PDIA3P1 under normoxic and hypoxic conditions in U251, U87MG, and A172 cell lines and P3 primary GBM cells. **e**–**h** PDIA3P1 expression in tumor samples classified by tumor grade (**e**, **g**) and transcriptome subtype (**f**, **h**) according to the TCGA and CGGA databases. **i**–**l** Kaplan–Meier survival curves of patients with high and low PDIA3P1 expression in GBMs (**i**, **k**) and LGGs (**j**, **l**) according to the TCGA and CGGA databases. Data are shown as the mean ± standard error of three independent experiments. Statistical significance was determined using Student’s *t* test, one-way ANOVA test and log-rank analysis. (*P < 0.05; **P < 0.01 and ***P < 0.001).

To identify the hypoxia related pseudogenes, we first used the microarray dataset of normoxic and hypoxic U87MG glioblastoma cells line (GSE45301) from GEO to generate differentially expressed pseudogenes. Here, we found 41 upregulated pseudogenes in hypoxia-treated U87MG cells (Fig. 1a, b). After filtering out the low expressing genes according to GSE45301 and TCGA datasets, we found that PDIA3P1 was highly expressed and obviously increased in hypoxia-treated glioma cells, as well as the tumor samples according to TCGA-LGG and TCGA-GBM datasets. To confirm that, RT-qPCR was performed to evaluate PDIA3P1 expression in normal human astrocytes (NHA), the glioma cell lines U251, U87MG, A172, and the primary P3 GBM cell line cultured under normoxic and hypoxic conditions. Results showed that glioma cell lines possessed higher PDIA3P1 levels than NHA cells. In addition, the GBM cell lines U87MG, A172, and P3 showed higher expression levels than the low-grade glioma (LGG) U251 cell line (Fig. 1c). In addition, hypoxia increased PDIA3P1 expression in all 4 glioma cell lines, as was expected (Fig. 1d).

To assess the clinical significance of PDIA3P1 in glioma patients, we evaluated the correlation between PDIA3P1 expression and patient clinicopathologic characteristics using the TCGA database. As shown in Table 1, high PDIA3P1 expression correlated with higher age (*p* < 0.001), WHO grade IV (Fig. 1e) and MES subtype (Fig. 1f), genomic alterations like IDH wild-type (*p* < 0.001), MGMT promoter non-methylation (*p* < 0.001), TERT promoter mutation (*p* < 0.001) and ATRX wild-type (*p* < 0.001), transcriptome score like low ABSOLUTE purity (*p* = 0.049), high ESTIMATE stromal score (*p* < 0.001), and immune score (*p* < 0.001). Expression and clinical profiles acquired from the CGGA database also confirmed our conclusions (Fig. 1g, h). These results indicated that high PDIA3P1 levels usually occurred in gliomas of high malignancy and were accompanied by infiltration of stromal and immune cells. Consistent with previous results, survival analysis demonstrated that both LGG and GBM patients with higher PDIA3P1 levels had shorter overall survival times than those with lower levels, according to the TCGA and CGGA databases (Fig. 1i–l). These findings suggested that PDIA3P1 was upregulated under hypoxic conditions and resulted in a poor prognosis in glioma patients.

**Table. 1:** Correlation between PDIA3P1 and clinicopathological characteristics in 608 gliomas.

Variable		PDIA3P1 high	PDIA3P1 low	*p* Value
Age	>45	205	111	<0.001
	≤45	103	189	
Gender	Male	189	166	0.131
	Female	119	134	
KPS	≥80	159	148	0.053
	<80	44	24	
WHO grade	II	77	139	<0.001
	III	79	158	
	IV	148	7	
Transcriptome subtype	Proneural (PN)	77	160	<0.001
	Classical (CL)	71	15	
	Mesenchymal (MES)	80	17	
IDH status	Wild-type	191	44	<0.001
	Mutant	139	286	
1p_19q Codeletion	Non-codeletion	249	243	0.349
	Codeletion	78	90	
MGMT promoter status	Unmethylated	112	49	<0.001
	Methylated	190	283	
TERT promoter status	Wild-type	38	124	<0.001
	Mutant	83	72	
ATRX status	Wild-type	281	181	<0.001
	Mutant	45	149	
ABSOLUTE purity	≥0.8	138	110	0.049
	<0.8	146	163	
ESTIMATE stromal score	≥600	205	118	<0.001
	<600	127	213	
ESTIMATE immune score	≥1000	195	130	<0.001
	<1000	137	201	

### PDIA3P1 promoted migration and invasion of glioma cells in vitro and in vivo

**Fig. 2:**
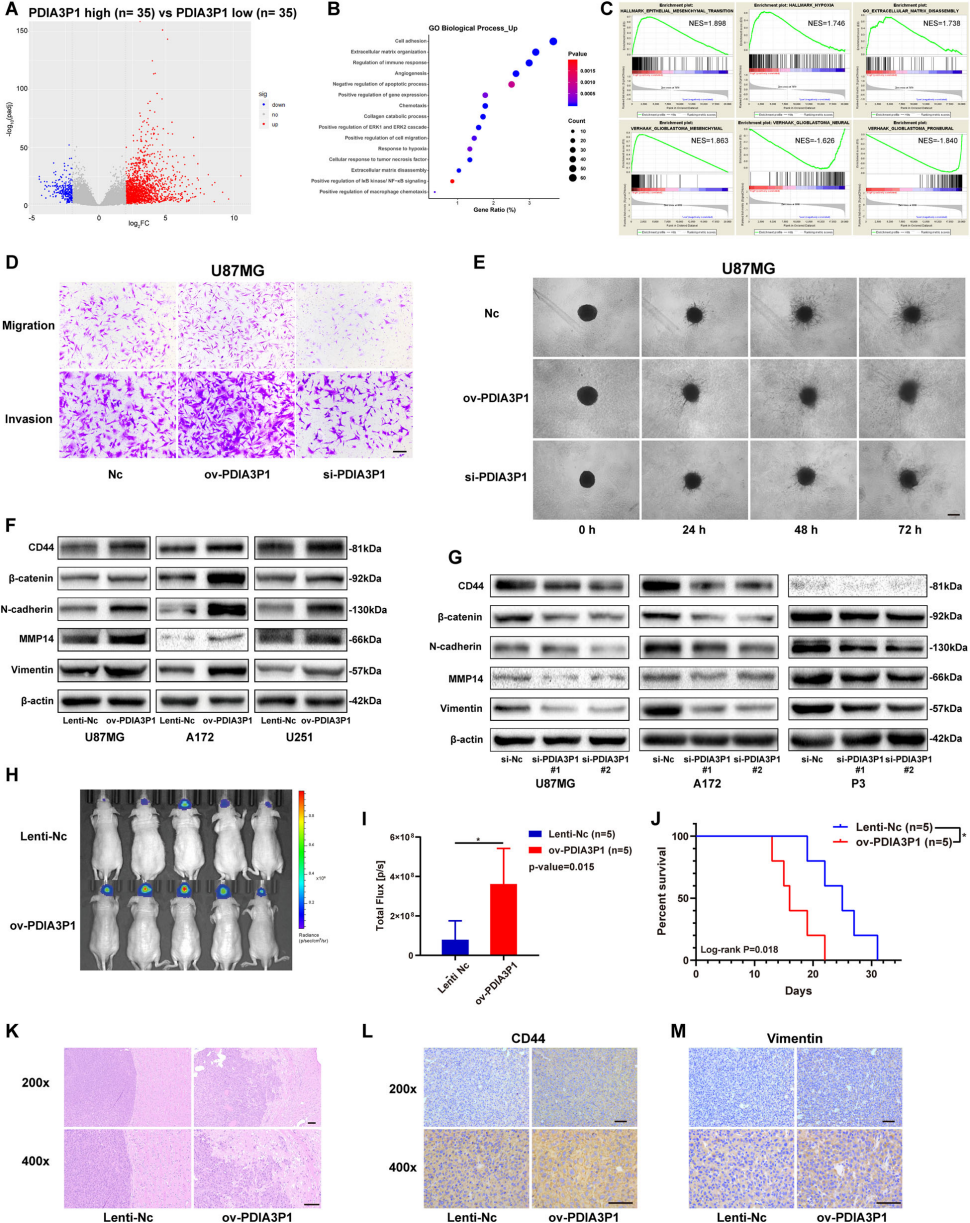
PDIA3P1 promoted migration and invasion of glioma cells in vitro and in vivo. **a** Volcano plot showing the differentially expressed genes between high-PDIA3P1 expressing patients (*n* = 35) and low-PDIA3P1 expressing patients (*n* = 35) according to the TCGA-GBM database. **b** Biological process enrichment analysis of upregulated genes in **a**. Positively correlated processes are listed on the *y*-axis. **c** GSEA confirming that high PDIA3P1 was associated with hypoxia and epithelial-mesenchymal transition (EMT), while low PDIA3P1 was associated with neural and proneural subtypes. **d** Migration and invasion capacity of U87MG cells transfected with control lentivirus (Lenti-Nc) or PDIA3P1 overexpressing lentivirus (ov-PDIA3P1), and siRNAs were assessed using transwell assay. Representative photographs are shown, scale bar: 100 μm. **e** 3D tumor spheroid invasion assay of U87MG cells transfected with lentivirus and siRNA. Representative images at 0 h, 24 h, 48 h, and 72 h are shown, scale bar: 200 μm. **f** Protein level of MES markers in U87MG, A172, and U251 cells transfected with the lentivirus overexpressing a control sequence or PDIA3P1 were assessed by western blotting. β-actin was used as control for normalization. **g** Protein level of MES markers in U87MG, A172 and P3 cells transfected with si-Nc and si-PDIA3P1 were assessed by western blotting. β-actin was used as control for normalization. **h** In vivo bioluminescent imaging analysis of tumor growth in xenograft nude mice at day 10. **i** Quantification of luminescent signals in the Lenti-Nc and ov-PDIA3P1 mice. **j** Survival analysis of nude mice orthotopically implanted with U87MG cells transfected with lentivirus overexpressing the control sequence or PDIA3P1. (*P* = 0.018 by log-rank analysis; data from 5 animals/group). **k** H&E staining of xenograft sections from PDIA3P1 overexpressing or negative control U87MG cell tissues on the same day of execution, scale bar: 100 μm. **l**, **m** Protein levels of CD44 (**l**) and Vimentin (**m**) in xenograft sections from PDIA3P1 overexpressing or negative control U87MG cell tissues were determined by immunohistochemistry (IHC) staining, scale bar: 50 μm. Data are shown as the mean ± standard error of three independent experiments. Statistical significance was determined using Student’s t test and log-rank analysis. (*P < 0.05; **P < 0.01 and ***P < 0.001).

To investigate the biological role of PDIA3P1 in glioma, we analyzed 70 samples from the top (*n* = 35) and bottom (*n* = 35) according to their PDIA3P1 level in the TCGA-GBM database. Differentially expressed genes (DEGs) were generated and results showed 1700 significantly upregulated genes and 250 significantly downregulated genes (Fig. 2a). Then, we conducted enrichment analysis of the DEGs to determine the biological processes that PDIA3P1 may modulate. As shown in Fig. 2b, high PDIA3P1 level was related to processes such as extracellular matrix disassembly, cell migration, and hypoxia. Moreover, GSEA analysis of 70 enrolled samples also confirmed that high PDIA3P1 expression was correlated with epithelial-mesenchymal transition (EMT), hypoxia, and extracellular matrix disassembly. In addition, we found that PDIA3P1 upregulation was positively correlated with the MES subtype and had a negative correlation with PN and NE signatures, according to the gene sets built by Verhaak (Fig. 2c). As the results suggest, PDIA3P1 may promote migration and invasion of GBMs. Therefore, we knocked down PDIA3P1 in U87MG and A172 cells and P3 primary GBM cells, and overexpressed PDIA3P1 in U251, U87MG, and A172 cells (Supplementary Fig. 1c, d). Transwell assay showed that PDIA3P1 overexpression promoted the migration and invasion capacity of glioma cells, while PDIA3P1 knockdown inhibited the capacity (Fig. 2d, Supplementary Fig. 1e–i). Similarly, high PDIA3P1 levels in U87MG cells resulted in a larger invasion area in the 3D tumor spheroid invasion assay (Fig. 2e) and a longer migration distance in U251 cells in the wound healing assay (Supplementary Fig. 1j). Next, we referred to the EMT gene set of GSEA and selected CD44, β-catenin, N-cadherin, Vimentin, and MMP14 as five representative markers to test their protein levels in vitro (Supplementary Fig. 1a). As shown in Fig. 2f, PDIA3P1 upregulation increased the protein level of MES markers, whereas PDIA3P1 knockdown inhibited their expression in glioma cells (Fig. 2g).

To determine the function of PDIA3P1 in vivo, luciferase-labeled U87MG cells stably overexpressing the control sequence or PDIA3P1 were injected into the brains of nude mice. Bioluminescence imaging 10 days after injection showed that mice bearing PDIA3P1 overexpressing U87MG cells exhibited significantly higher tumor viability than control mice (Fig. 2h, i). As expected, increased PDIA3P1 levels in U87MG cells also resulted in shorter overall survival in xenograft mice (Fig. 2j). To assess tumor properties in vivo, we euthanized an additional 5 mice in each group at day 10 following injection. H&E staining showed obscure tumor borders and tenuous ECM in PDIA3P1 overexpressing samples, however, samples in the control group exhibited a clear tumor border and dense ECM (Fig. 2k). Similarly, immunohistochemistry (IHC) assays demonstrated a higher level of CD44 and Vimentin in high-PDIA3P1 xenograft samples compared to the control groups (Fig. 2l, m). These results confirmed that PDIA3P1 promotes glioma MES transition in vitro and in vivo, as is represented by the enhanced migration and invasion capacities of tumor cells.

### PDIA3P1 functioned as a ceRNA to sponge miR-124-3p in gliomas

**Fig. 3:**
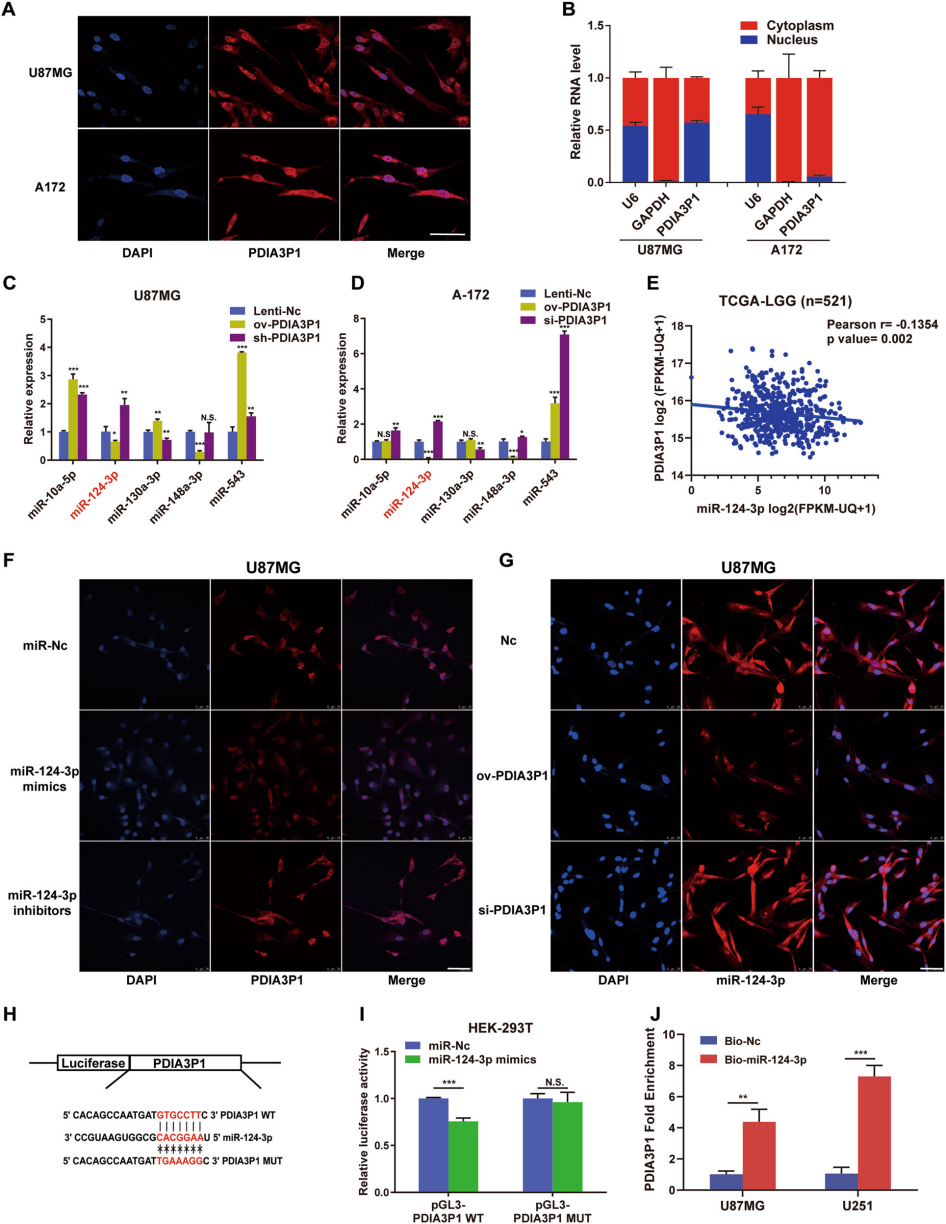
PDIA3P1 functioned as a microRNA sponge to combine with miR-124-3p. **a** Fluorescence In Situ Hybridization (FISH) was used to detect the location of PDIA3P1 in U87MG cells and A172 cells, scale bar: 50 μm. **b** Relative PDIA3P1 expression in the cytoplasm and nuclei of U87MG and A172 cells was determined by RT-qPCR. **c**, **d** Relative expression of the top 5 predicted microRNA in PDIA3P1 overexpressing and knockdown U87MG cells (**c**) and A172 cells (**d**). Titles marked red on the *x*-axis represent a significant decrease in expression in the ov-PDIA3P1 group and a significant increase in the si-PDIA3P1 group (*P* < 0.05). **e** Correlation between the expression of miR-124-3p and PDIA3P1 in low grade glioma was determined using the TCGA-LGG datasets. **f** FISH assay using a PDIA3P1 probe to test its expression in miR-124-3p overexpressing and knockdown U87MG cells. Representative images are shown, scale bar: 50 μm. **g** FISH assay using a miR-124-3p probe to test its expression in PDIA3P1 overexpressing and knockdown U87MG cells. Representative images are shown, scale bar: 50 μm. **h** Construction of wild type (WT) and mutant type (MUT) luciferase reporter vectors based on the predicted binding site of miR-124-3p in PDIA3P1. **i** U87MG cells were co-transfected with the reporter vectors and miR-124-3p or miR-Nc. Luciferase activity was assessed 48 h after transfection. **j** U87MG cells and U251 cells were transfected with biotinylated miR-124-3p (Bio-miR-124-3p) or biotinylated NC (Bio-Nc). 48 h after transfection, cells were collected for a biotin-based pulldown assay. PDIA3P1 expression levels were analyzed by RT–qPCR. Data are shown as the mean ± standard error of three independent experiments. Statistical significance was determined using Student’s t test, one-way ANOVA test and Pearson correlation test. (*P < 0.05; **P < 0.01; ***P < 0.001; N.S. indicates no significant difference).

We first performed FISH and subcellular fractionation to determine PDIA3P1 distribution in glioma cells. Similar to the study in OSCC^25^, results showed that PDIA3P1 was mainly localized in the cytoplasm in U87MG and A172 cells (Fig. 3a, b), indicating that it may function as a ceRNA to sponge microRNAs and regulate the expression of target genes at the posttranscriptional level^28^. Next, we predicted potential microRNAs to which PDIA3P1 might bind using the online tool Starbase^29^. As the TCGA-GBM database lacks microRNA expression profiles, we utilized the TCGA-LGG database to filter target microRNAs. Among all the statistically relevant microRNAs, we screened MES-related microRNAs and selected the top 5 in terms of their expression level in clinical samples, including miR-10a, miR-124-3p, miR-130a-3p, miR-148a-3p, and miR-543. RT-qPCR was performed to verify their expression in PDIA3P1 overexpressing or knockdown U87MG and A172 cells. Result showed that only miR-124-3p expression was decreased in PDIA3P1 overexpressing glioma cells and increased in the knockdown counterparts (Fig. 3c, d). In addition, correlation analysis demonstrated a significant negative correlation between miR-124-3p and PDIA3P1 expression (Fig. 3e), suggesting that miR-124-3p may be a direct target of PDIA3P1. Next, FISH assay confirmed the negative correlation between PDIA3P1 and miR-124-3p in U87MG, A172, and U251 cells under normoxic or hypoxic conditions (Fig. 3f, g, Supplementary Fig. 2a–f). To further confirm our results, dual luciferase reporter plasmids were constructed which contained the wildtype (WT) or mutant type (MUT) sequences based on the putative miR-124-3p binding site in PDIA3P1 (Fig. 3h). As expected, miR-124-3p overexpression significantly suppressed luciferase activity in HEK-293T cells transfected with the WT plasmids, but this was abolished by the mutation of the predicted binding site (Fig. 3i). In addition, a biotin-labeled pulldown assay was used to determine whether miR-124-3p could interact with PDIA3P1. Figure 3j showed that the RNA level PDIA3P1 was significantly increased in the miR-124-3p pull-down product isolated from U87MG and U251 cells. Collectively, these results showed that PDIA3P1 is enriched in the cytoplasm and directly sponges miR-124-3p in glioma cells.

### MiR-124-3p targeted RELA and suppressed MES transition of glioma cells in vitro

**Fig. 4:**
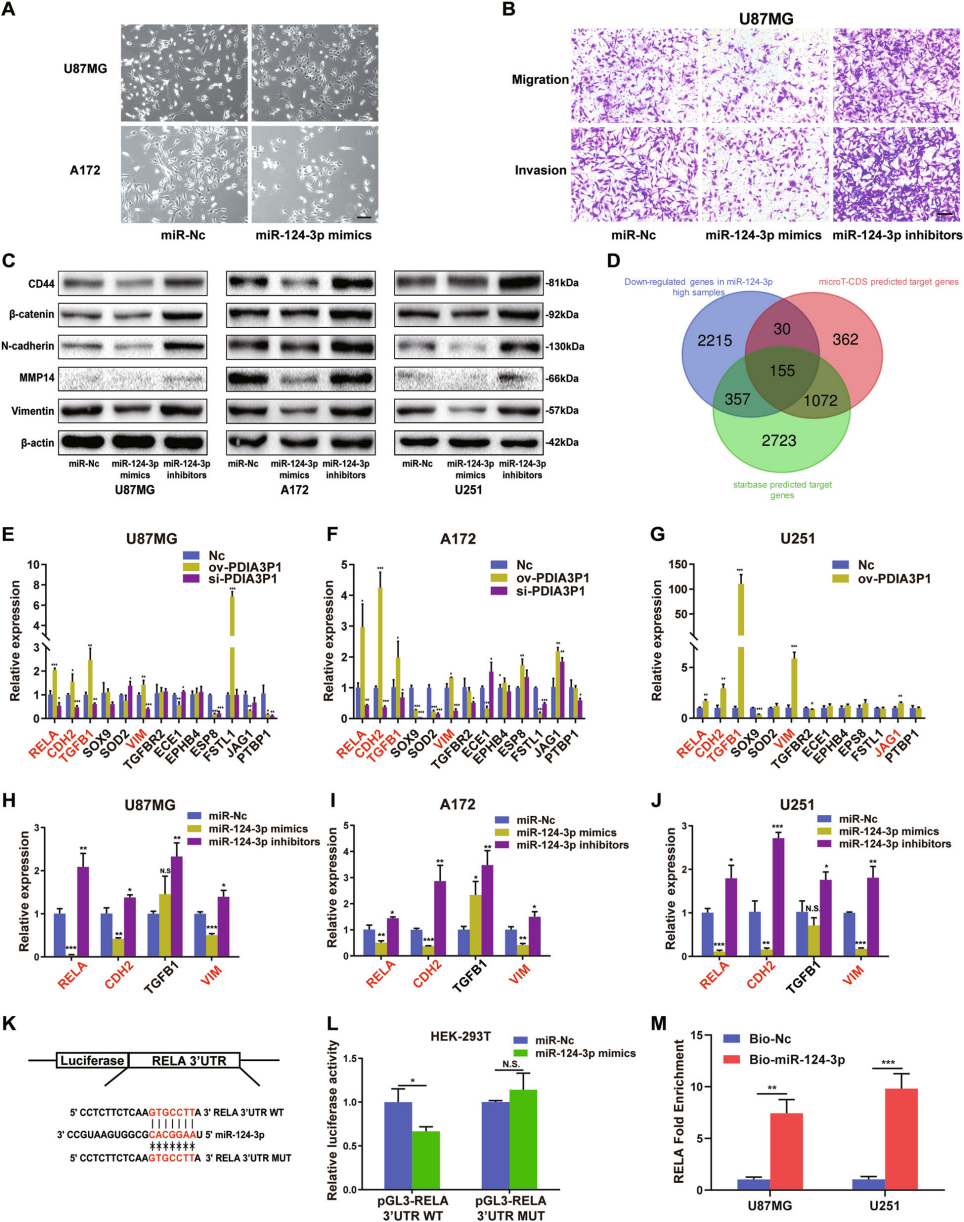
MiR-124-3p targeted RELA and suppressed MES transition of glioma cells in vitro. **a** Morphological changes of U87MG cells and A172 cells after transfection with miR-Nc or miR-124-3p mimics, scale bar: 100 μm. **b** Migration and invasion of U87MG cells transfected with miR-Nc, miR-124-3p mimics, and miR-124-3p inhibitors was assessed using transwell assay. Representative photographs are shown, scale bar: 100 μm. **c** Protein levels of MES markers in U87MG, A172, and U251 cells transfected with miR-Nc, miR-124-3p mimics, and miR-124-3p inhibitors were assessed by western blotting. β-actin was used as a control for normalization. **d** A Venn diagram depicting 155 common potential targets of miR-124-3p as predicted by microT-CDS, Starbase, and data analysis according to the TCGA-LGG dataset. **e**–**g** Relative expression of the possible targets predicted in **d** associated with MES transition was assessed by RT-qPCR in PDIA3P1 overexpressing and knockdown U87MG (**e**), A172 (**f**), and U251 (**g**) cells. Titles marked red on the x-axis represent a significant increase in expression in the ov-PDIA3P1 group and a decrease in the si-PDIA3P1 group. **h**–**j** Relative expression of the potential targets of miR-124-3p verified in **e**–**g** was assessed by RT-qPCR in miR-124-3p overexpressing and knockdown U87MG (**h**), A172 (**i**), and U251 (**j**) cells. Titles marked red the on *x*-axis represent a significant decrease in expression in the miR-124-3p mimics group and an increase in the miR-124-3p inhibitors group. **k** Construction of wild type (WT) and mutant type (MUT) luciferase reporter vectors based on the predicted binding site of miR-124-3p in the 3’ UTR of RELA. **l** U87MG cells were co-transfected with the reporter vectors and miR-124-3p or miR-Nc. Luciferase activity was assessed 48 h after transfection. **m** U87MG cells and U251 cells were transfected with biotinylated miR-124-3p (Bio-miR-124-3p) or biotinylated NC (Bio-Nc). 48 h after transfection, cells were collected for a biotin-based pulldown assay. RELA expression levels were analyzed by RT–qPCR. Data are shown as the mean ± standard error of three independent experiments. Statistical significance was determined using Student’s t test and one-way ANOVA test. (*P < 0.05; **P < 0.01; ***P < 0.001. N.S. indicates no significant difference).

Several studies have elucidated the tumor suppressive role of miR-124-3p in glioma^30,31^. First, we transfected miR-124-3p mimics into U87MG and A172 cells and observed obvious morphological changes in the miR-124-3p overexpressing cells. Cells became flat and pseudopods were shortened compared to the control cells. Moreover, the previously scattered glioma cells aggregated and adhered to each other when miR-124-3p was upregulated (Fig. 4a). Transwell assay also demonstrated that miR-124-3p overexpression inhibited the migration and invasion capacity of U87MG and A172 cells, while miR-124-3p knockdown facilitated these attributes (Fig. 4b, Supplementary Fig. 3a–c). Moreover, the protein levels of CD44, β-catenin, N-cadherin, Vimentin, and MMP14 were also decreased in miR-124-3p overexpressing glioma cells and increased in miR-124-3p deprived cells (Fig. 4c, Supplementary Fig. 3d). Next, we combined the target genes predicted by Starbase and microT-CDS with downregulated genes in high-miR-124-3p expressing samples using the TCGA-LGG database (Fig. 4d). Results revealed 155 common genes in total, and among these we screened out 13 MES-related genes in glioma through consulting published research. Then, the expression of 13 potential targets was tested using RT-qPCR in PDIA3P1 overexpressing or knockdown U87MG and A172 cells, and PDIA3P1 overexpressing U251 cells. Results showed that only the expression of RELA, CHD2, TGFB1, and VIM exhibited the expected changes (Fig. 4e–g). Next, we overexpressed or inhibited miR-124-3p in U87MG, A172, and U251 cells and evaluated the expression of RELA, CHD2, TGFB1, and VIM. As shown in Fig. 4h–j, the expression of RELA, CDH2, and VIM were suppressed by miR-124-3p mimics and increased by the miR-124-3p inhibitors. Alternatively, TGFB1 expression was unexpectedly increased in miR-124-3p overexpressing U87MG and A172 cells so it was excluded from further research. RELA, which encodes the NF-κB p65 subunit, is known as an upstream molecule in the induction of glioma MES transition. Thus, we determine to verify the target effect of RELA prior to CDH2 and VIM. First, we identified a significant negative correlation between the expression of miR-124-3p and RELA (Supplementary Fig. 3e). Next, dual luciferase reporter plasmids were constructed which contained the WT and MUT 3’UTR of RELA, based on the putative miR-124-3p binding site (Fig. 4k). As shown in Fig. 4l, miR-124-3p suppressed luciferase activity in the WT plasmid, but had little effect in the MUT plasmid. In addition, biotin-labeled pulldown assay showed that the RNA level of RELA was markedly elevated in the miR-124-3p pull-down product compared to the control in U87MG and U251 cells (Fig. 4m). Suggesting that miR-124-3p interacts with RELA and inhibited glioma MES transition through impeding its activity.

### PDIA3P1 induced glioma MES transition by activating the NF-κB pathway

**Fig. 5:**
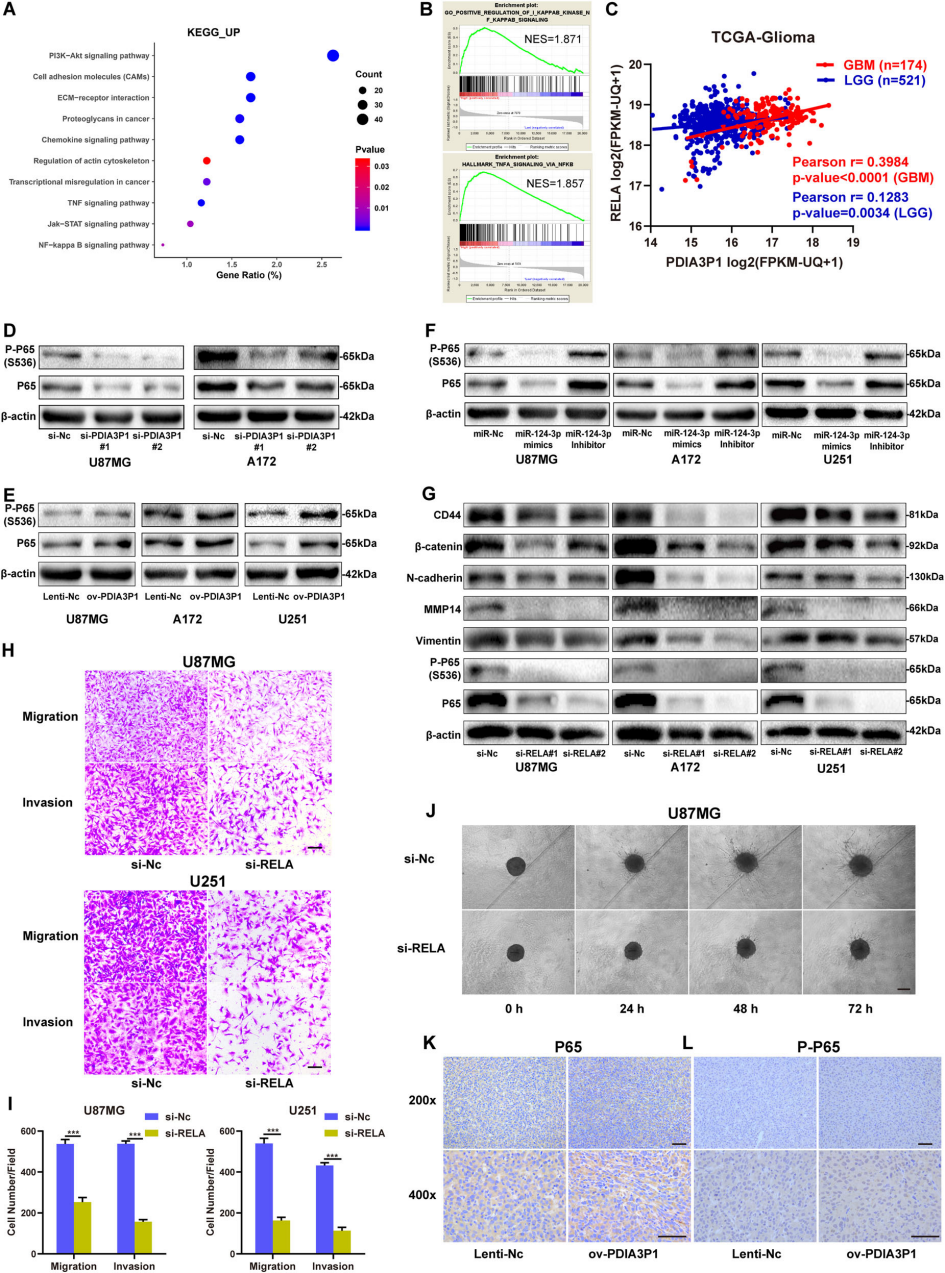
PDIA3P1 induced glioma MES transition by activating the NF-κB pathway. **a** KEGG pathway enrichment analysis of upregulated genes in Fig. 2A. Positively correlated processes were listed on the *y*-axis. **b** GSEA confirmed that high PDIA3P1 expression is associated with the NF-κB pathway. **c** Correlation between the expression of PDIA3P1 and RELA in LGG and GBM was determined using TCGA datasets. **d** Protein levels of P65 and phosphorylated P65 in the NF-κB pathway in U87MG and A172 cells transfected with si-Nc and si-PDIA3P1 were assessed by western blotting. **e** Protein levels of P65 and phosphorylated P65 in the NF-κB pathway in U87MG, A172, and U251 cells transfected with a lentivirus overexpressing the control sequence or PDIA3P1 were assessed by western blotting. **f** Protein levels of P65 and phosphorylated P65 in the NF-κB pathway in U87MG, A172, and U251 cells transfected with miR-Nc, miR-124-3p mimics, and miR-124-3p inhibitors were assessed by western blotting. **g** Protein levels of MES markers, P65, and phosphorylated P65 in the NF-κB pathway in U87MG, A172, and U251 cells transfected with si-Nc and si-RELA were assessed by western blotting. β-actin was used as a control for normalization. **h**, **i** Migration and invasion capacity of U87MG and U251 cells transfected with si-Nc and si-RELA was assessed using a transwell assay. Representative photographs are shown, scale bar: 100 μm. **j** 3D tumor spheroid invasion assay of U87MG cells transfected with si-Nc and si-RELA. Representative images at 0 h, 24 h, 48 h, and 72 h are shown, scale bar: 200 μm. **k**, **l** Protein levels of P65 (**k**) and P-P65 (**l**) in xenograft sections from PDIA3P1 overexpressing or negative control U87MG cell tissues were determined by IHC staining, scale bar: 50 μm. Data are shown as the mean ± standard error of three independent experiments. Statistical significance was determined using Student’s *t* test. (*P < 0.05; **P < 0.01; ***P < 0.001).

NF-κB transcriptional program plays a critical role in glioma MES transition. Our findings verified that RELA is a direct target of miR-124-3p, suggesting that PDIA3P1 might induce glioma MES transition by activating the NF-κB pathway. As shown in Fig. 5a, b, both KEGG enrichment and GSEA analysis demonstrated that the NF-κB pathway was activated in high-PDIA3P1 samples. In addition, a positive correlation between the expression of PDIA3P1 and RELA was confirmed in glioma samples (Fig. 5c). To further consolidate our results, the protein level of NF-κB p65 and phosphorylated p65 (S536) were determined by western-blotting in PDIA3P1 and miR-124-3p conditioned cell models. Results confirmed that PDIA3P1 knockdown or miR-124-3p overexpression inhibited the protein level of p65 and its phosphorylated form, while PDIA3P1 overexpression or miR-124-3p inhibition increased their expression in our glioma cell models (Fig. 5d–f, Supplementary Fig. 4b). Considering the possibility that PDIA3P1 may activate the NF-κB pathway and promote glioma MES transition through increasing RELA expression, we knocked down RELA using small interfering RNAs (siRNAs) in U87MG, A172, and U251 cells and determined the protein levels of CD44, β-catenin, N-cadherin, Vimentin, and MMP14. As expected, RELA knockdown significantly inhibited NF-κB activity and the downstream protein levels of MES markers in glioma cells (Fig. 5g). Further, the results of the transwell assay showed that RELA knockdown indeed suppressed the migration and invasion capacity of U87MG and U251 cells (Fig. 5h, i). In addition, the tumor spheroid invasion assay showed that the invasion area was decreased in RELA-inhibited U87MG cells (Fig. 5j). To confirm our findings in vivo, we performed IHC assay to determine the protein levels in xenograft tumor samples. As shown in Fig. 5k, the NF-κB p65 and phosphorylated p65 (S536) were higher in PDIA3P1 overexpressing samples, indicating that PDIA3P1 promotes glioma MES transition by activating the NF-κB pathway in vitro and in vivo.

### The HIF-1α and HIF-1β heterodimer transcriptionally activated PDIA3P1

**Fig. 6:**
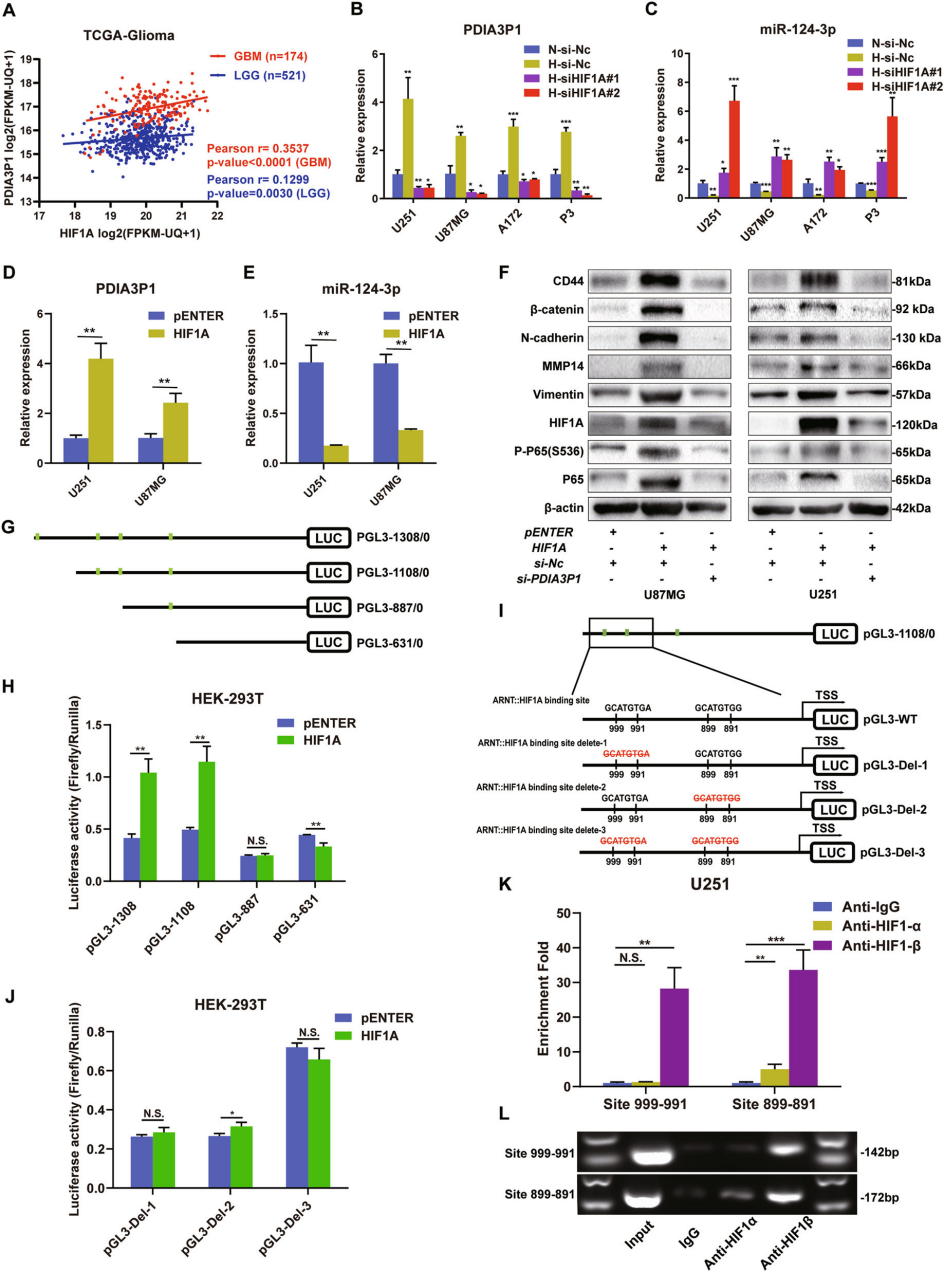
The HIF-1α and HIF-1β heterodimer binds to the hypoxia response element (HRE) of PDIA3P1 promoters to facilitate its expression. **a** Correlation between the expression of HIF1A and PDIA3P1 in LGG and GBM was determined using the TCGA datasets. **b** Relative PDIA3P1 expression was determined by RT-qPCR in U251, U87MG, A172, and P3 cells transfected with si-Nc and si-HIF1A and cultured under hypoxic and normoxic conditions. **c** Relative miR-124-3p expression was determined by RT-qPCR in U251 and U87MG cells transfected with si-Nc and si-HIF1A, cultured under hypoxic and normoxic conditions. **d** Relative expression of PDIA3P1 in U251 and U87MG cells transfected with control plasmids (pENTER) and plasmids overexpressing HIF1A under hypoxic conditions. **e** Relative expression of miR-124-3p in U251 and U87MG cells transfected with control plasmids (pENTER) and plasmids overexpressing HIF1A under hypoxic conditions. **f** Protein levels of MES markers, HIF1A, P65, and phosphorylated P65 in the NF-κB pathway in U87MG and U251 cells with the indicated treatments. **g** Schematic diagram of luciferase reporter constructs of a 1308 base pair (bp) region upstream of PDIA3P1 transcription start site (TSS) and three truncated mutation plasmids according to the predicted the binding sites (marked in green). **h** Dual luciferase assay in HEK-293T cells showed HIF1A overexpression increased promoter activity in PGL3-1308 and PGL3-1108. **i** Schematic diagram of luciferase reporter constructs containing the PDIA3P1 promoter (PGL3-WT) and deletion construct (PGL3-Del-1/2/3) in which the presumed HIF1A::ARNT binding site was deleted. **j** Dual luciferase assay in HEK-293T cells showed HIF1A overexpression did not increase promoter activity in PGL3-Del-1 and PGL3-Del-3 transfected cells, and only a slight increase occurred in PGL3-Del-2 transfected cells. **k** ChIP-qPCR analysis indicated higher fold enrichment of promoter site 999-991 and site 899-891 in the anti-HIF-1β antibody group compared to the IgG group in U251 cells, indicating that HIF-1β can directly bind the PDIA3P1 promoter. **l** ChIP-PCR assay showed that HIF-1β directly interacted with the predicted sites within the PDIA3P1 promoter in U251 cells. A specific, strong band of the expected size was detected in the input DNA and chromatin complex precipitated by anti-HIF-1β. No band was detected in the chromatin complex precipitated by the antibody against IgG, and a weak band was detected in site 899-891 in the chromatin complex precipitated by anti-HIF-1α. Data are shown as the mean ± standard error of three independent experiments. Statistical significance was determined using Student’s t test and one-way ANOVA test. (*P < 0.05; **P < 0.01; ***P < 0.001. N.S. indicates no significant difference).

**Fig. 7:**
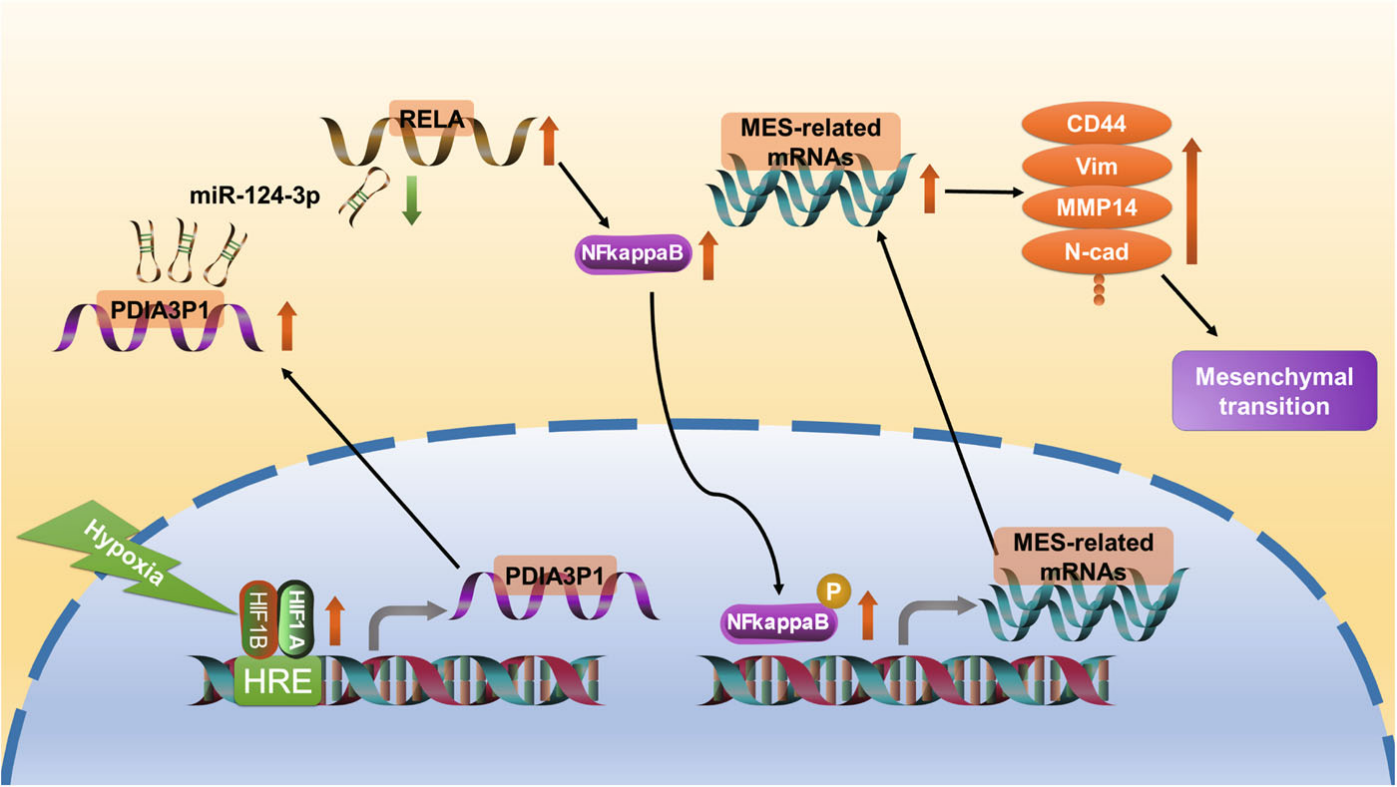
Schematic model of hypoxia-induced PDIA3P1 promotes glioma MES transition via PDIA3P1-miR-124-3p-RELA axis. Hypoxia-induced factor 1 directly bound to the promotor region and upregulated the expression of PDIA3P1, a lncRNA which promotes glioma MES transition via sponging miR-124-3p and activating downstream NF-κB pathway.

HIF1A is the most important transcriptional factor in tumor survival under hypoxic conditions. First, we analyzed the correlation between the expression level of HIF1A and PDIA3P1. Results showed an apparent positive correlation in GBM samples and a slight but still significant positive correlation in LGG samples (Fig. 6a). Next, we knocked down HIF1A and determined the expression levels of PDIA3P1 and miR-124-3p in glioma cells. PDIA3P1 upregulation in hypoxia-cultured glioma cells was reversed when HIF1A was inhibited (Fig. 6b). Alternatively, miR-124-3p expression was significantly increased in HIF1A knockdown hypoxia-treated glioma cells (Fig. 6c). Then, U251 and U87MG cells were transfected with pENTER or plasmids overexpressing HIF1A and maintained under hypoxic conditions. Result showed that HIF1A overexpression increased PDIA3P1 levels, and miR-124-3p was suppressed accordingly (Fig. 6d, e). In addition, western-blot assay verified that HIF1A overexpression activated the NF-κB pathway and its downstream MES markers, however this effect was suppressed or even reversed by PDIA3P1 inhibition in U87MG and U251 cells (Fig. 6f). To investigate whether HIF1A regulated PDIA3P1 expression through binding to its HRE on the promoter region, A~1300-base pair (bp) region upstream of the transcription start site (TSS) and three truncated mutation plasmids were constructed according to the predicted the binding sites (Fig. 6g). Plasmids were transfected into HEK-293T cells and results showed that HIF1A overexpression increased the relative luciferase activity in pGL3-1308/0 and pGL3-1108/0 transfected cells but did not elevate the activity in pGL3-887/0 and pGL3-631/0 transfected cells (Fig. 6h). Moreover, no significant decrease in luciferase activity was observed with promotor deletion from 1308 to 1108, indicating that the binding region located between −1108 and −887 is the functional HRE of PDIA3P1. As there were two putative binding sites located between −1108 and −887, three plasmids were constructed containing the deletion of site 999-991 (pGL3-Del-1), site 899-891 (pGL3-Del-2), and both sites (pGL3-Del-3) (Fig. 6i). Deletion plasmids were co-transfected with HIF1A-overexpressing plasmids or control vector into HEK-293 cells. Results showed that the increase of relative luciferase activity was abolished in the HIF1A overexpressing pGL3-Del-1 group and the pGL3-Del-3 group, yet only slight enhancement was found in the pGL3-Del-2 group, but the level was far less than that of pGL3-1108/0 (Fig. 6j). This result indicates that both sites might be functional HRE on the PDIA3P1 promoter. To confirm that, ChIP assay was performed. DNA fragments were collected from hypoxia-cultured U251 cells and immunoprecipitated using anti-IgG, anti-HIF-1α and anti-HIF-1β. RT-qPCR assay showed an approximate 30-fold enrichment of both promoter amplicons in the anti-HIF-1β group, while no obvious enrichment of site 999-991 and only about an 8-fold enrichment of site 899–891 was observed in the anti-HIF-1α group (Fig. 6k), and this was confirmed by agarose gel electrophoresis (AGE) of PCR products (Fig. 6l), suggesting that the HIF-1α and HIF-1β heterodimer can directly bind to the HRE in the PDIA3P1 promotor region to facilitate its expression (Fig. 7).

## Discussion

Accumulating evidence has demonstrated that lncRNA plays an important role in carcinogenesis and malignant tumor progression^32^. Considering the large scale of lncRNAs in the human transcriptome and the high heterogeneity of gliomas, the functional landscape of lncRNAs in human glioma remain elusive. Since the hypothesis of ceRNA was developed in 2011^33^, an increasing number of studies have been published to decode the sophisticated RNA network^28,34^. In 2017, Kong et al. found that PDIA3P1 knockdown decreased the migration, invasion, and proliferation of HCC cells by affecting the p53 pathway, but the detailed mechanisms were not studied in detail^24^. In other studies, Sun and colleagues demonstrated that PDIA3P1 is overexpressed in OSCC and promotes tumor cell proliferation by absorbing miR-185-5p^25^. In this study, we found that lncRNA PDIA3P1 was mainly localized in the cytoplasm, indicating that PDIA3P1 may function as an endogenous microRNA sponge. Further investigation revealed that miR-124-3p is a novel target of PDIA3P1. Several studies have demonstrated the antitumor effects of miR-124-3p in multiple cancers^35,36^. Moreover, Zhang et al. found that miR-124-3p overexpression inhibited tumor growth and angiogenesis by targeting NRP-1 in glioblastoma^31^. In addition, Bhaskaran and colleagues constructed a module overexpressing miR-124-3p and two other microRNAs to explore new therapeutic strategies against GBM by targeting EZH2^30^. In this study, we also found that miR-124-3p inhibited the malignant characteristics of glioma cells, as was reflected in the impaired migration and invasion capacity and decreased expression of MES markers. Then, we verified that RELA, a proto-oncogene which is also known as the NF-κB P65 subunit, was a novel target of miR-124-3p.

In 2013, Bhat et al. found that PN GSCs transformed to the MES state through the NF-κB pathway, with an elevated expression of CD44 and radioresistant phenotypes^37^. Thereafter, several studies were conducted to elucidate the potential mechanisms of how gliomas acquire the MES-signature through activating NF-κB transcriptional programs^38–41^. Furthermore, therapeutic approaches targeting the NF-κB pathway in GBM have also been investigated with some success^42,43^. Considering its vital role in MES gliomas and the devastating consequences of resistance to multiple therapies, finding new strategies to deactivate the NF-κB system is essential to improving clinical outcomes for glioma patients^37^.

Jin et al. revealed that hypoxia is crucial to GSC maintenance and correlates with the MES-signature in glioblastoma^5^. Therefore, the mechanisms behind hypoxia-induced MES transition in glioma is of interest. Rius et al. demonstrated that NF-κB is a critical transcriptional activator of HIF-1α. In turn, NF-κB activation required the accumulation of the HIF-1α protein under hypoxic conditions^44^. There is also evidence showing that NF-κB promotes the transcription and activity of HIF-1α in several cancers^45,46^. In addition, interventions aimed at HIF-1α consistently lead to the simultaneous inactivation of the NF-κB pathway^47^. Further, researchers found that COMM domain-containing 1 (COMMD1) disrupted the dimerization of HIF-1α and HIF-1β, which then inhibited NF-κB mediated gene expression and tumor cell invasion^48^. However, the detailed mechanisms of how HIF-1α affects the activity of NF-κB during tumor progression are still largely unknown. In this study, we revealed that HIF-1 heterodimer mediated PDIA3P1 upregulation, and miR-124-3p downregulation promoted the MES transition of gliomas through activating the NF-κB pathway. This effect was partly reversed by RELA knockdown, indicating that PDIA3P1 is an important connection between hypoxia and the NF-κB pathway.

In summary, we revealed that hypoxia-induced factor 1 directly bound to the promotor region and upregulated the expression of PDIA3P1, which promotes cell migration and invasion through the PDIA3P1-miR-124-3p-RELA axis in human gliomas. These findings identified PDIA3P1 as a novel link between hypoxia and NF-κB-mediated glioma MES transition, suggesting it as a promising candidate for prognosis and molecular target in glioma patients.

## Supplementary information

Supplementary Figure 1

Supplementary Figure 2

Supplementary Figure 3

Supplementary Figure 4

Supplementary Figure Legends

Supplementary Table 1

Supplementary Table 2

Supplementary Table 3
