# Supplementary figures and images for: Hypoxia-induced lncRNA PDIA3P1 promotes mesenchymal transition via sponging of miR-124-3p in glioma
Source: Cell Death Dis. 2020 Mar 3;11(3):168. doi: 10.1038/s41419-020-2345-z (PMC7054337; doi:10.1038/s41419-020-2345-z)

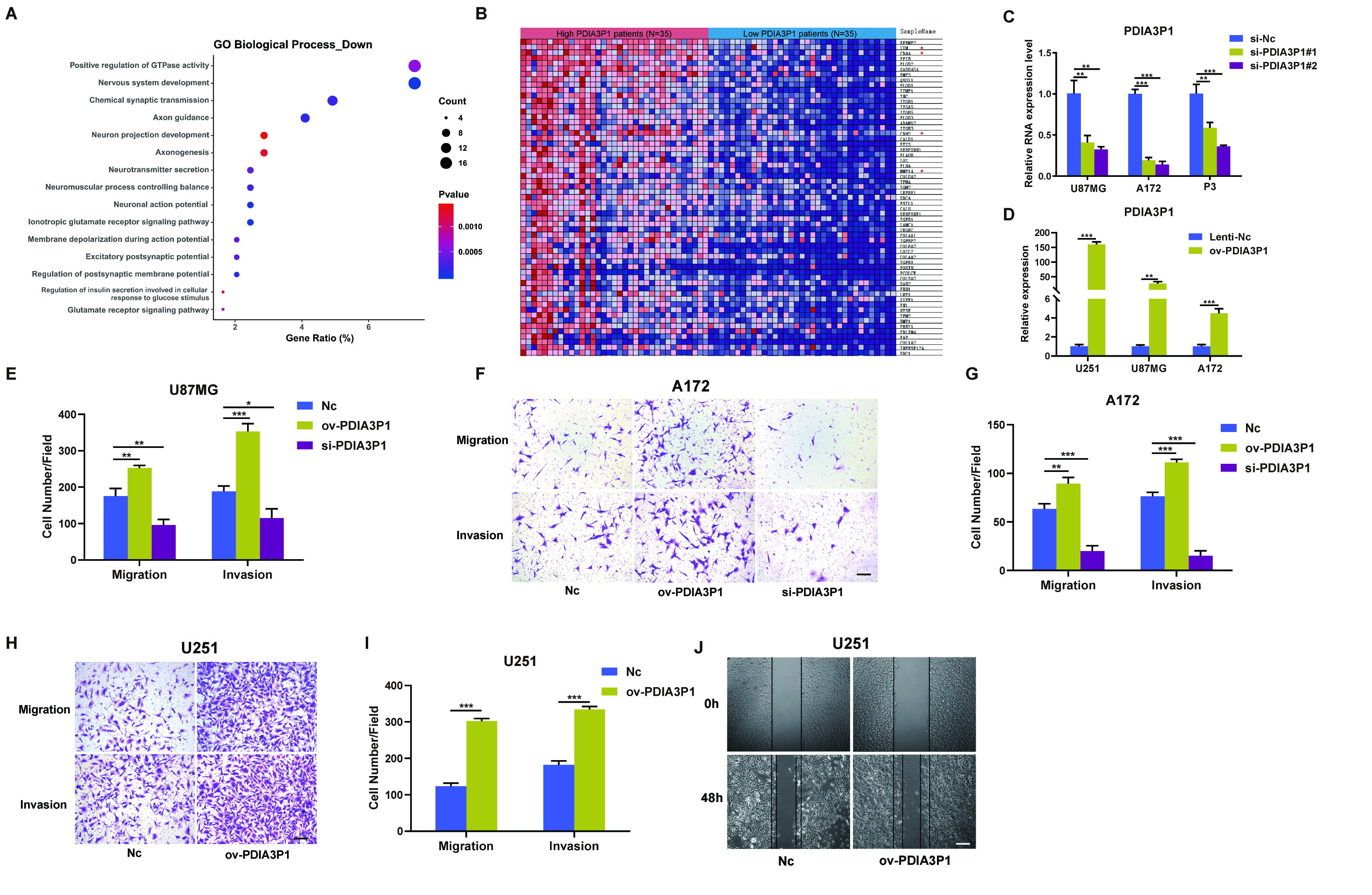

Supplement: Supplementary file 1 — Supplementary Figure 1 [file 41419_2020_2345_MOESM1_ESM.tif]

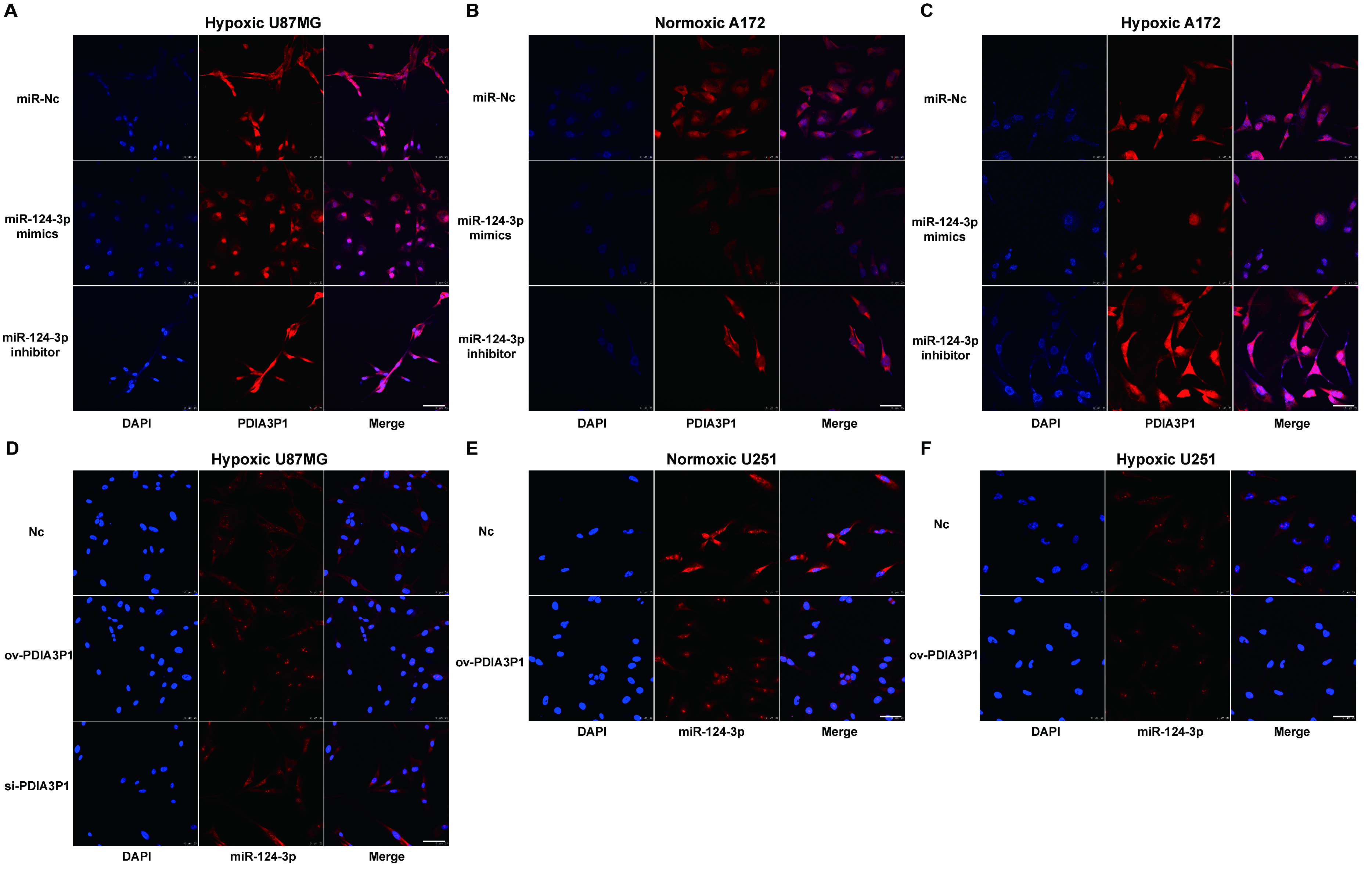

Supplement: Supplementary file 2 — Supplementary Figure 2 [file 41419_2020_2345_MOESM2_ESM.tif]

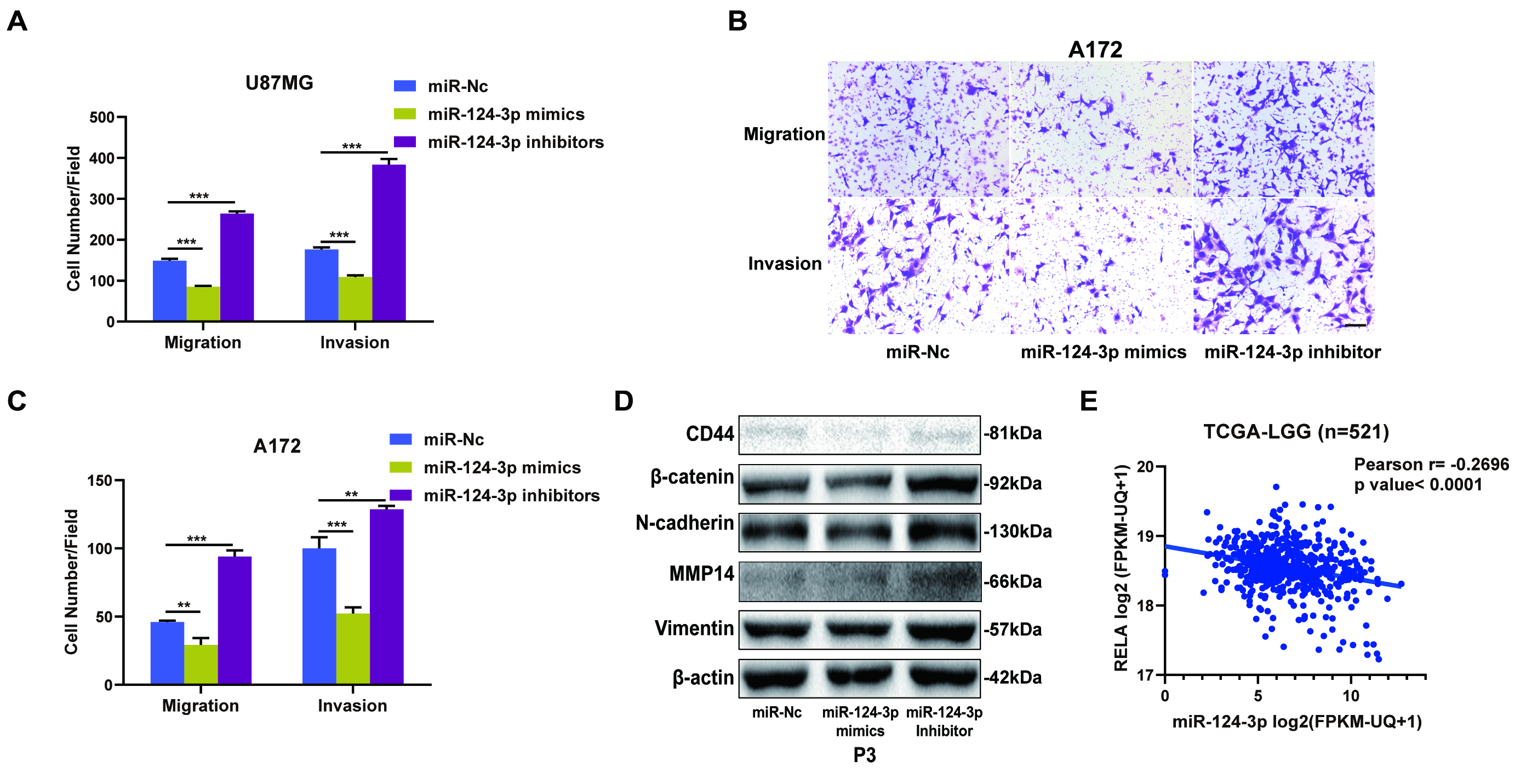

Supplement: Supplementary file 3 — Supplementary Figure 3 [file 41419_2020_2345_MOESM3_ESM.tif]

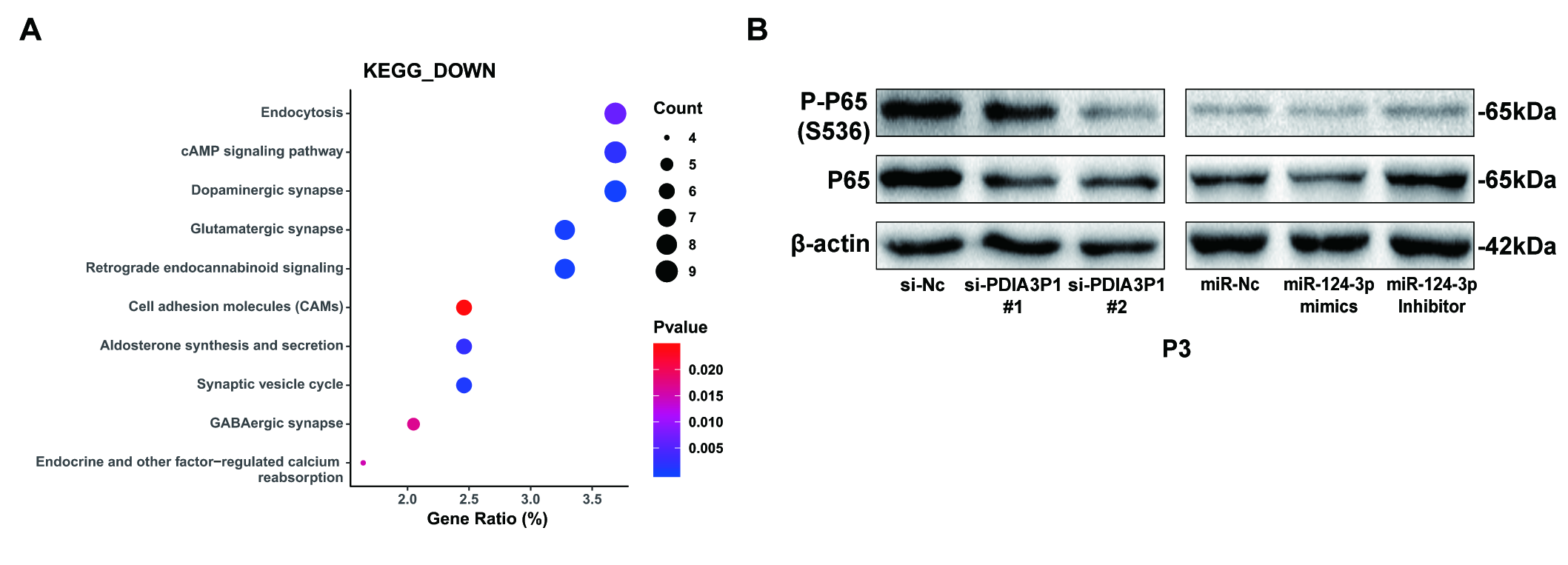

Supplement: Supplementary file 4 — Supplementary Figure 4 [file 41419_2020_2345_MOESM4_ESM.tif]
