# Supplementary material for: Hypoxia-induced lncRNA PDIA3P1 promotes mesenchymal transition via sponging of miR-124-3p in glioma
Source: Cell Death Dis. 2020 Mar 3;11(3):168. doi: 10.1038/s41419-020-2345-z (PMC7054337; doi:10.1038/s41419-020-2345-z)
Supplement: Supplementary file 5 — Supplementary Figure Legends [file 41419_2020_2345_MOESM5_ESM.docx]

Supplementary Figure Legends

Figure S1. PDIA3P1 promotes migration and invasion of glioma cells in vitro.

(A) Biological process enrichment analysis of downregulated genes high-PDIA3P1 samples which showed that a low PDIA3P1 level in GBM patients mainly correlated with neural development and synaptic transmission. Negatively correlated processes were listed on left y-axis. (B) Heatmap showed the upregulated genes in high-PDIA3P1 samples enriched in EMT gene set of GSEA. (C) PDIA3P1 knockdown efficiency using U87MG, A172 and P3 cells. (D) PDIA3P1 overexpression efficiency using lentivirus stably transfectd in U251, U87MG and A172 cells. (E) Quantification of transwell assay showed migration and invasion capacities in PDIA3P1 conditioned U87MG cells. (F, G) Migration and invasion capacity of U87MG cells transfected with lentivirus and siRNA altering PDIA3P1 level were assessed using transwell assay. Representative photographs were shown, scale bar: 100 μm. (H, I) Migration and invasion capacity of U251 cells transfected with lentivirus overexpressing control sequence and PDIA3P1 were assessed using transwell assay. Representative photographs were shown, scale bar: 100 μm. (J) Wound healing assay demonstrated the enhanced migration capacity in PIDA3P1 overexpressing U251 cells, scale bar: 200 μm. Data are shown as the mean ± standard error (S.E.) of three independent experiments. Statistical significance was determined using Student’s t test and one-way ANOVA test. (*P < 0.05; **P < 0.01; ***P < 0.001. N.S. indicates no significant difference.)

****Figure S2. FISH assay determined the negative correlation between PDIA3P1 and miR-124-3p.****

(A) FISH assay using probe to detect PDIA3P1 expression in miR-124-3p conditioned hypoxia-cultured U87MG cells. (B) FISH assay using probe to detect PDIA3P1 expression in miR-124-3p conditioned normoxia-cultured A172 cells. (C) FISH assay using probe to detect PDIA3P1 expression in miR-124-3p conditioned hypoxia-cultured A172 cells. (D) FISH assay using probe to detect miR-124-3p expression in PDIA3P1 conditioned hypoxia-cultured U87MG cells. (E) FISH assay using probe to detect miR-124-3p expression in PDIA3P1 conditioned normoxia-cultured U251 cells. (F) FISH assay using probe to detect miR-124-3p expression in PDIA3P1 conditioned hypoxia-cultured U251 cells, scale bar: 50 μm.

Figure S3. miR-124-3p suppress glioma MES transition in vitro.

(A) Quantification of transwell assay showed migration and invasion capacities in miR-124-3p conditioned U87MG cells. (B, C) Migration and invasion capacity of A172 cells transfected with miR-124-3p mimics or miR-124-3p inhibitor were assessed using transwell assay. Representative photographs were shown, scale bar: 100 μm. (D) Protein level of MES markers in miR-124-3p overexpressing or knockdown P3 cells. (E) Correlation between the expression of miR-124-3p and RELA in low grade glioma was determined using the TCGA-LGG datasets. Data are shown as the mean ± standard error (S.E.) of three independent experiments. Statistical significance was determined using Student’s t test, one-way ANOVA test and Pearson correlation test. (*P < 0.05; **P < 0.01; ***P < 0.001. N.S. indicates no significant difference.)

Figure S4. PDIA3P1 induces glioma MES transition by activating NF-κB pathway.

(A) KEGG pathway enrichment analysis of downregulated genes in high-PDIA3P1 samples. Negatively correlated processes were listed on left y-axis. (B) Protein level of NF-κB p65 and phosphorylated p65 (S536) in PDIA3P1 knockdown and miR-124-3p conditioned P3 cells.
