# Supplementary material for: Hypoxia-induced lncRNA PDIA3P1 promotes mesenchymal transition via sponging of miR-124-3p in glioma
Source: Cell Death Dis. 2020 Mar 3;11(3):168. doi: 10.1038/s41419-020-2345-z (PMC7054337; doi:10.1038/s41419-020-2345-z)
Supplement: Supplementary file 6 — Supplementary Table 1 [file 41419_2020_2345_MOESM6_ESM.docx]

Supplementary Table S1. Sequence of primers for qRT-PCR

| Gene | Forward primer (5’-3’) | Reverse primer (5’-3’) |
| --- | --- | --- |
| PDIA3P1 | GGAAAACCACTGGGGAGGAC | CAGTGCAGCTAAGAAATGGCT |
| Site 999-991 | GGTACAAATAGGCTGCCAACTC | ATTGATGCCTCCTACCGTAACC |
| Site 899-891 | GGTTACGGTAGGAGGCATCAAT | TTGCTTCTGCTTGTCATTGCTT |
| GAPDH | GCACCGTCAAGGCTGAGAAC | TGGTGAAGACGCCAGTGGA |
| HIF1A | ATCACCCTCTTCGTCGCTTC | ACTTATCTTTTTCTTGTCGTTCGC |
| RELA | CTTCCAAGAAGAGCAGCGTG | TTTCGGTTCACTCGGCAGAT |
| CDH2 | CCTTTCAAACACAGCCACGG | TGTTTGGGTCGGTCTGGATG |
| TGFB1 | TTGACTTCCGCAAGGACCTC | CTCCAAATGTAGGGGCAGGG |
| SOX9 | GACTTCTGAACGAGAGCGAGA | CCGTTCTTCACCGACTTCCTC |
| SOD2 | CCGACCTGCCCTACGACTAC | TCTCCTCGGTGACGTTCAGG |
| VIM | AATGGCTCGTCACCTTCGTG | CAGAGAAATCCTGCTCTCCTCG |
| TGFBR2 | TTGGCGAGGAGTTTCCTGTT | GAGGGAAGCTGCACAGGAGT |
| ECE1 | GCACCTCCTCGAAAACTCCA | TCCTCGATCCTGGTCTCGTT |
| EPHB4 | GGTGACATTCCCTCAGGTGG | TGCACGTCACACACTTCGTA |
| ESP8 | ACAGTGGTGGCAGTATCGTG | CATCCTCTGGTGTGGAGTCG |
| FSTL1 | CAGACCACGATGTGGAAACGC | TGGTTGAGGTAGGTCTTGCC |
| JAG1 | TCACGGGAAGTGCAAGAGTC | GTTTCACAGTAGGCCCCCTC |
| PTPB1 | AGAACGCCCTAGTGCAGATG | GTTGCCGTAGTCCTTGGTCA |
| miR-10a-5p | ATGCCTTCATTTTACCCTGTAGAT | TATGGTTTTGACGACTGTGTGAT |
| miR-124-3p | GCCGCTAAGGCACGCG | TATGGTTGTTCACGACTCCTTCAC |
| miR-130a-3p | CGTCTCCAGTGCAATGTTAAAAG | TATGGTTTTGACGACTGTGTGAT |
| miR-148a-3p | AATCGAACCTTCAGTGCACTACA | TATGCTTGTTCTCGTCTCTGTGTC |
| miR-543 | AACTAAATTAAACATTCGCGGTG | TATGCTTGTTCTCGTCTCTGTGTC |
| U6 | ATTGGAACGATACAGAGAAGATT | GGAACGCTTCACGAATTTG |
