# Supplementary material for: Hypoxia-induced lncRNA PDIA3P1 promotes mesenchymal transition via sponging of miR-124-3p in glioma
Source: Cell Death Dis. 2020 Mar 3;11(3):168. doi: 10.1038/s41419-020-2345-z (PMC7054337; doi:10.1038/s41419-020-2345-z)
Supplement: Supplementary file 7 — Supplementary Table 2 [file 41419_2020_2345_MOESM7_ESM.docx]

****Supplementary Table S2. Sequences for siRNA, microRNA mimics and inhibitor****

| Name | Sequence (5’-3’) |
| --- | --- |
| si-Nc | UUCUCCGAACGUGUCACGUTT |
| si-PDIA3P1#1 | GCAAAGACCUGAAUAUCGUTT |
| si-PDIA3P1#2 | AGUCCUGAAUGACAACAAATT |
| si-RELA#1 | UCUUCCUACUGUGUGACAATT |
| si-RELA#2 | GGAGUACCCUGAGGCUAUATT |
| si-HIF1A#1 | CAAUCAAGAAGUUGCAUUATT |
| si-HIF1A#2 | UCGACUAUCUGCUCCAAGUUCTT |
| miR-Nc | CAGUACUUUUGUGUAGUACAA |
| miR-124-3p mimics | UAAGGCACGCGGUGAAUGCC |
| miR-124-3p inhibitor | CAGUACUUUUGUGUAGUACAA |
