# Supplementary material for: Hypoxia-induced lncRNA PDIA3P1 promotes mesenchymal transition via sponging of miR-124-3p in glioma
Source: Cell Death Dis. 2020 Mar 3;11(3):168. doi: 10.1038/s41419-020-2345-z (PMC7054337; doi:10.1038/s41419-020-2345-z)
Supplement: Supplementary file 8 — Supplementary Table 3 [file 41419_2020_2345_MOESM8_ESM.docx]

****Supplementary Table S3. FISH probe sequence.****

| Name | Sequence |
| --- | --- |
| PDIA3P1 | GCAAAGACCUGAAUAUCGU |
| miR-124-3p | TTGGCATTCACCGCGTGCCTTA |
